# The contractile efficiency of the mantle muscle of European common cuttlefish (*Sepia officinalis*) during cyclical contractions

**DOI:** 10.1242/jeb.249297

**Published:** 2024-11-08

**Authors:** Nicholas W. Gladman, Graham N. Askew

**Affiliations:** School of Biomedical Sciences, University of Leeds, Leeds, West Yorkshire LS2 9JT, UK

**Keywords:** Cephalopod, Work loop, Metabolism, Contractile efficiency

## Abstract

Escape jet propulsion swimming in cuttlefish (*Sepia officinalis*) is powered by the circular muscles surrounding the mantle cavity. This mode of locomotion is energetically costly compared with undulatory swimming. The energetic cost of swimming is determined by the mechanical power requirements and the efficiency with which chemical energy is transferred into useful mechanical work. One step in this energy transduction process is the transfer of energy from ATP hydrolysis into mechanical work by the muscles. Here, we determined the efficiency of this step, termed the contractile efficiency. Muscle preparations from the circular muscles of the mantle cavity were subjected to sinusoidal length changes at different cycle frequencies, and stimulated with a phase and duration that maximised initial net work. Changes in ATP, arginine phosphate and octopine content between control and exercised muscles were determined and used to calculate the energy released from ATP hydrolysis (*E*_met_). The maximum contractile efficiency (the ratio of net work to *E*_met_) was 0.37, occurring at the same cycle frequency at which mechanical power was maximal and that was used during jet propulsion swimming, suggesting that cuttlefish muscle is adapted to generate muscular power efficiently. The overall efficiency of cuttlefish jet propulsion swimming was estimated to be 0.17, which is broadly comparable to that measured during animal flight and human-powered pedalled locomotion, indicating the high energetic costs of jet propulsion swimming are not due to inefficient locomotion per se; instead, they result from the relatively high mechanical power requirements.

## Teaser

**Highlighted Article:** The contractile efficiency of the muscles that power escape jet propulsion swimming in cuttlefish is relatively high. Maximal efficiency and power output occur at the cycle frequency used *in vivo*.

## INTRODUCTION

Cephalopod molluscs can propel themselves during swimming by jet propulsion, drawing water into their mantle cavity and ejecting it via the funnel. Jet propulsion swimming is energetically expensive compared with undulatory swimming (Bartol et al., 2008; Wells and O'Dor, 1991), and this has been attributed to the theoretically lower efficiency resulting from expelling relatively high velocity jets comprising relatively small volumes of fluid (Alexander, 2002; Gladman and Askew, 2023). However, investigations into the fluid motions in the wake have demonstrated that whole-cycle propulsive efficiency in cephalopod molluscs can be relatively high (in excess of 0.85; Anderson and Grosenbaugh, 2005; Bartol et al., 2009a,b; Gladman and Askew, 2023; Neil and Askew, 2018) and higher than those of undulatory swimmers (Maertens et al., 2015); therefore, another explanation for the relatively high energetic cost of jet propulsion swimming in cephalopod molluscs is required.

**Fig. 1.**
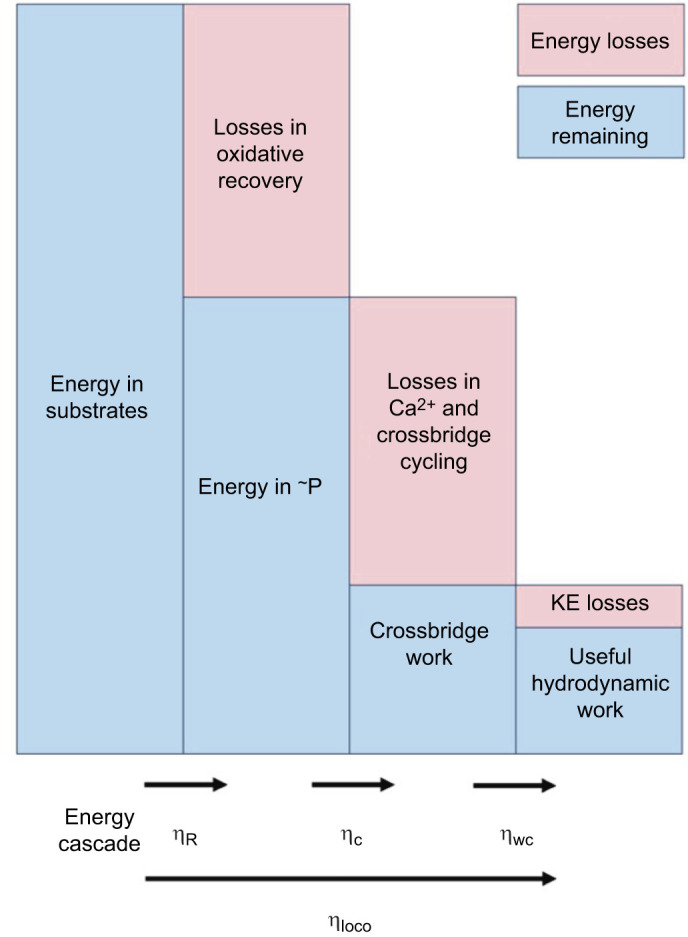
**Energy cascade during swimming.** At each step, the energy remaining is indicated in a blue box and the energy losses in a pink box. The oxidative recovery efficiency (η­_R_) is the efficiency with which energy in substrates (e.g. glycogen) is converted into high-energy (∼P) phosphates (e.g. ATP). The contractile efficiency (η­_c_) is the efficiency with which energy in ∼P is utilised by the crossbridges to generate mechanical work; at this step, energy losses are associated with Ca^2+^ and crossbridge cycling. The whole-cycle efficiency (η_wc_) is the efficiency with which work done by the crossbridges is transferred to the water to overcome drag, with losses being associated with the kinetic energy (KE) transferred to the wake and done during refilling (Gladman and Askew, 2023). The overall efficiency of locomotion (η­_loco_) is the efficiency with which chemical energy in the substrates is transferred into useful hydrodynamic work: η­_loco_=η­_R_×η­_c_×η_wc_.

The overall efficiency of locomotion (η­_loco_) is the efficiency with which chemical energy in the substrates that fuel the activity is used by the locomotory muscles and transferred into useful work in the environment (i.e. useful hydrodynamic work in the case of swimming); it is the ratio of the mechanical power output to the metabolic power input. In common with all modes of locomotion, η­_loco_ in jet propulsion swimming is determined by the efficiency of a number of underlying steps (Fig. 1; Askew et al., 2010): (1) the oxidative recovery efficiency (η_R_) with which high-energy phosphates (e.g. ATP) are produced by mitochondrial oxidative phosphorylation from chemical substrates (e.g. glycogen); (2) the contractile efficiency (η­_c_) with which mechanical work is generated by the actomyosin interaction during the crossbridge cycle, utilising chemical energy derived from the hydrolysis of high-energy phosphates (e.g. ATP); and (3) whole-cycle propulsive efficiency (η_wc_) with which work performed by the crossbridges is transferred to the environment, and accounts for the kinetic energy losses associated with the acceleration of fluid during the refilling phase of jet propulsion swimming in cuttlefish (see eqn 2 in Gladman and Askew, 2023). At each stage within this energy cascade, energy may be lost through the inefficient transduction of energy from one step to the next. Hence, the overall efficiency of locomotion is given by:
(1)

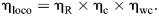
The circumferentially arranged, fast-twitch muscle fibres [termed central mitochondria-poor (CMP) fibres; Preuss et al., 1997] of the mantle musculature provide much of the power during jet propulsion swimming in cephalopod molluscs; here, we quantified their contractile efficiency. These muscle fibres are fuelled anaerobically (Grieshaber and Gäde, 1976), with ATP being resynthesised though the dephosphorylation of arginine phosphate (ArgP) to arginine (Arg; via the arginine kinase pathway: ADP+ArgP ⇌ ATP+Arg), leading to the formation of octopine to maintain redox balance in the musculature; octopine is formed from arginine and pyruvate (via the octopine dehydrogenase pathway: Arg+pyruvate+NADH ⇌ octopine+NAD^+^+H_2_O; Portner et al., 1996; Storey and Storey, 1979). During both hypoxia and exhaustive exercise in adult European cuttlefish (*Sepia officinalis*) and bay scallops (*Argopecten irradians*), octopine is accumulated, while ArgP and ATP are depleted in the locomotory muscles (Chih and Ellington, 1983; Storey and Storey, 1979), with similar changes occurring in whole-animal and nerve-stimulated muscle preparations (Grieshaber and Gäde, 1976).

In order to quantify muscle contractile efficiency, energy utilisation and mechanical work generation must both be measured, ideally simultaneously. Whilst the mechanical performance of cephalopod muscle has been quantified (Gladman and Askew, 2022; Kier and Curtin, 2002; Milligan et al., 1997; Rosenbluth et al., 2010; Thompson et al., 2014, 2023; Zullo et al., 2022), the simultaneous metabolic energy expenditure has not, and therefore the contractile efficiency of cephalopod mollusc muscle is currently unknown. The aim of this study was to determine the contractile efficiency of the mantle musculature of the European common cuttlefish (*S. officinalis*) during cyclical contractions which simulate jet propulsion swimming, in order to gain insights into the determinants of the high metabolic cost of transport in jet-propelled swimmers. We also investigated the relationship between mechanical power output, contractile efficiency and cycle frequency, and hypothesised that cycle frequency would impact contractile efficiency, with cycle frequencies observed *in vivo*, 1.39 Hz during escape responses (Gladman and Askew, 2022), expected to be the most efficient and to also generate the highest power output. The mechanical work that the muscles generate was measured during cyclical contractions using the work loop technique (Josephson, 1985), and the metabolic energy expenditure was determined by quantifying the changes in the concentrations of high-energy phosphates and end products of anaerobic metabolism (Feala et al., 2007; Lowry and Passonneau, 1972; Smith et al., 2005).

## MATERIALS AND METHODS

### Animals

Juvenile European common cuttlefish (*Sepia officinalis* Linnaeus 1758) were reared from eggs taken as by-catch upon fishing gear by R. K. Stride, Christchurch, Dorset, UK, during May 2016 in the English Channel. Eggs and hatchlings were maintained as previously described (Gladman and Askew, 2022). Juvenile animals were housed in two recirculating artificial saltwater systems [Aqua One Reef synthetic, Kong's (Aust.) Pty Ltd, Sydney, NSW, Australia] of 358 l (length×width×height; 91×69×57 cm). The temperature and salinity were maintained at 15±1°C and 32±1 PSU during the first 3 months after animals hatched; this temperature was then gradually decreased (over 10 days) to 11±1°C. Animals were fed bi-daily on alternate days using live river shrimp (*Palaemon varians*; Aquatic Live fish foods, Woodford, London, UK). Cuttlefish were reared for 12 months under these conditions, with final group sizes consisting of up to 30 animals per tank.

Water quality was monitored to ensure suitable ranges were maintained; temperature and salinity were monitored twice-daily; pH (7.8–8.1) and nitrogenous compounds were monitored monthly (NH_4_ ≤0.5 mg l^−1^; NO_2_ ≤0.2 mg l^−1^; NO_3_ ≤80 mg l^−1^); 25% water changes were carried out twice per week. The maintained parameters for temperature and salinity fell within the natural range of animals in the English Channel (Cefas, 2012).

### Muscle dissection

Prior to muscle dissection, cuttlefish (*N*=25) were euthanised in accordance with schedule 1 (Appendix D) of the UK Animals (Scientific Procedures) Act 1986 (amended 2012); animals were chilled to 4°C in artificial seawater before the brain, vertical and optical lobes were destroyed by pithing. Prior use of sedative or anaesthetic was avoided because of the distress and adverse reactions reported by its administration in cephalopods (Andrews et al., 2013; Roumbedakis et al., 2020). Muscle preparations were then dissected out in artificial seawater at 11±0.5°C from the central zone of the ventral mantle wall, approximately 40% of the mantle length measured from the tip of the ventral mantle. Dissected muscle preparations were pared down to select the central zone of the muscle (muscle preparation mass 125.4±7.3 mg; optimal length *L*_0_ 14.65±0.43 mm).

### Muscle mechanical performance

A stainless-steel metal ring was attached to each end of the muscle preparation using suture thread (2-0 USP, black braided silk non-absorbable, non-sterile surgical suture, LOOK surgical specialities corporation, Reading, PA, USA). These stainless-steel rings were used to attach the preparation to a fixed hook at one end of a Perspex flow-through muscle chamber, and the arm of an ergometer (Aurora Scientific Dual-mode lever system model 300B-LR, Aurora Scientific Inc., Aurora, ON, Canada). Approximately 500 ml of artificial seawater at 11±0.5°C was recirculated through the muscle chamber.

Muscles were activated using 0.2 ms pulses delivered using a stimulus isolation unit (UISO model 236, Hugo Sachs Elektronik, March, Germany) via parallel platinum wire electrodes that ran the length of the muscle preparation. Muscle length was increased incrementally by 0.5 mm, and a series of isometric twitches were used to optimise length, to that which yielded the highest active twitch force (*L*_0_). Using twitches rather than tetani to optimise length may mean that muscle preparations were not at the length that yields maximum tetanic force or maximum net work (e.g. the length for maximum twitch force is approximately 4% shorter than that for maximum tetanic force: see Thompson et al., 2014; however, in vertebrates a different relationship has been reported with the optimum length for maximum tetanic force and net work being shorter than that for twitches: see Askew and Marsh, 1997; Holt and Azizi, 2014). Muscle preparations (*N*=25) were subjected to sinusoidal cyclical length changes, symmetrically about *L*_0_ using a strain amplitude of 0.075 *L*_0_, at one of three frequencies: [0.8 Hz (*N*=9), 1.4 Hz (*N*=8) or 2 Hz (*N*=8)] using the work loop technique (Josephson, 1985). The range of cycle frequencies selected encompassed the jet cycle frequency observed during swimming (1.39 Hz; Gladman and Askew, 2022). To ensure metabolite changes could be detected, muscles were subjected to either 15 or 30 cycles: at cycle frequencies of 0.8 and 1.4 Hz, muscle preparations were subjected to 15 cycles; at 2 Hz, 30 cycles were used as force and net work continued to increase up to the 15th cycle (see Fig. S1). A stimulation frequency of 50 Hz (pulse width 0.2 ms) was used and the timing and duration of stimulation were set to those previously found to maximise net power output (Gladman and Askew, 2022; see Table S1).

Instantaneous power was determined as the product of the muscle's velocity and the force generated. Cycle-by-cycle net power was determined as the average of the instantaneous power over the cycle. Net work per cycle (the difference between the work done by the muscle during shortening and the work done on the muscle during lengthening) was calculated from the ratio of net power to cycle frequency. Total net work was calculated as the sum of the cycle-by-cycle net work. Muscle mass-specific work and power were calculated by dividing the work and power values by the blotted wet mass of the muscle preparation. The average net work that was used in the calculation of contractile efficiency was calculated as the mean over the 15 or 30 cycles. The net power output in cycles 3 and 4 in this study and in a previous study (Gladman and Askew, 2022; *N*=5) are compared in Fig. S2.

### Muscle energetics

Following the cyclical contractions, muscles were immediately removed from the experimental chamber, weighed (blotted wet mass) and snap frozen in liquid nitrogen before being stored at −80°C. The weighing and freezing process took <30 s in total, with recovery expected to be minimal over this time [recovery of the phosphagen pool following exercise is slow in cuttlefish, with the process taking between 150 and 200 min (recovery rate of 0.05–0.06 μmol g^−1^ min^−1^ at 15°C) to return to levels recorded at rest; Melzner et al., 2006]. Each muscle preparation was paired with a control, non-exercised sample from the same animal. These control samples were treated in the same way as the experimental preparations. A total of 25 preparations (1 preparation per animal) were used, each being utilised for a single bout of cyclical contractions at one frequency: 0.8 Hz (*N*=8), 1.4 Hz (*N*=8) and 2 Hz (*N*=9).

Metabolites were extracted from tissues using a modification of the methods of Gäde et al. (1978). Frozen muscle preparations were placed in 2 ml micro tubes (Eppendorf, Hamburg, Germany) and homogenised in 6% perchloric acid (Honeywell Fluka, Honeywell International Inc., Morris Plains, NJ, USA) in 40% ethanol (Sigma-Aldrich, St Louis, MO, USA; ratio of one part muscle to two parts solution) maintained at −20°C for 20 min using a bead mill homogeniser (Qiagen TissueLyser LT, Qiagen, Hilden, North Rhine-Westphalia, Germany). The homogenised sample was transferred to 1.5 ml micro tubes and centrifuged at 10,000 ***g*** for 30 min at room temperature (Sigma 1-14 Microfuge, Sigma Laborzentrifugen GmbH, Osterode am Harz, Niedersachsen, Germany). The supernatant was collected and neutralised with 0.125 volumes 3 mol l^−1^ potassium carbonate (Fisher Chemical, Pittsburgh, PA, USA) in 50 mmol l^−1^ MES buffer (Alfa Aesar, Haverhill, MA, USA). Neutralised samples were centrifuged again at 10,000 ***g*** for 15 min and the final supernatant collected and stored at −80°C.

#### Measurement of ATP

The ATP content of samples was determined following Lowry and Passonneau (1972), through a two-step reaction, catalysed by hexokinase (HK) and glucose 6-phosphate dehydrogenase (G6PD):

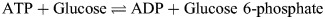
(catalysed by HK),

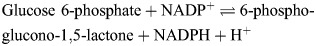
(catalysed by G6PD).

NADPH absorbs light at 340 nm, with the increase in absorbance being used to determine the content of ATP from the stoichiometry of the reactions.

The reaction mixture consisted of 25 mmol l^−1^ Tris-HCl (Lonza, Castleford, West Yorkshire, UK), 1 mmol l^−1^ magnesium chloride (Fluorochem Ltd, Hadfield, Derbyshire, UK), 0.5 mmol l^−1^ dithiothreitol (DTT; Fluorochem Ltd), 1 mmol l^−1^ glucose (Sigma-Aldrich), 0.5 mmol l^−1^ NADP (Merck Chemicals Ltd, Darmstadt, Germany) and 50 µl neutralised homogenate in a final volume of 2 ml. The reaction was initiated by the simultaneous addition of 1 unit of G6PD and 1 unit of HK (Sigma-Aldrich). Prior to the addition of G6PD and HK, the absorbance was read at 340 nm, 1 cm light path (Ultrospec 2100 Pro UV/Visible spectrophotometer, Biochrom, Harvard Bioscience Inc., Holliston, MA, USA); 20 min after the addition of G6PD and HK, absorbance was re-read. A blank (cuvette containing the reaction solutions and 50 µl deionised water rather than muscle homogenate) was included in all runs as a background control, which was deducted from all measurements to correct for any absorbance associated with the reaction solutions and the cuvette itself.

#### Measurement of arginine phosphate

The content of arginine phosphate in the muscle preparations was determined following Mira de Orduña (2001). Arginine content of samples was first determined through a three-step reaction, facilitated by the action of arginase, urease and glutamate dehydrogenase:

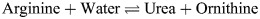
(catalysed by arginase),

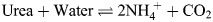
(catalysed by urease),



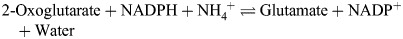
(catalysed by glutamate dehydrogenase).

NADPH absorbs light at 340 nm, with the decline in absorbance being used to determine the content of arginine from the stoichiometry of the reactions.

ArgP content was subsequently determined using the same reaction pathways, with ArgP dephosphorylated using 1 mol l^−1^ hydrochloric acid (following Morris and Adamczewska, 2002; Fisher Chemical, Loughborough, Leicestershire, UK). Hydrochloric acid was added in the same ratio as muscle homogenate (i.e. 50 µl) and samples were heated for 90 s at 100°C. Samples were cooled before 50 µl of 1 mol l^−1^ sodium hydroxide (Fisher Chemical) was added to neutralise the solution.

Reactions were carried out using the l-arginine assay kit (K-large, Megazyme Ltd, Wicklow, Ireland). Each assay was performed at room temperature, using the neutralised homogenate in a buffered solution [containing triethanolamine (TEA), polyvinylpolypyrrolidone (PVPP), α-ketoglutaric acid (α-KG), ADP, orthophosphoric acid, 2-oxoglutarate and sodium azide], and containing 0.10 ml 0.13 mmol l^−1^ NADPH. Prior to any enzyme addition, the solution was gently mixed and left for 2 min; the absorbance was then measured at 340 nm, 1 cm light path using an Ultrospec 2100 Pro UV/Visible spectrophotometer (Biochrom, Harvard Bioscience Inc.). Next, 10 µl (7.8 U ml^−1^) of the glutamate dehydrogenase solution was added, the cuvette inverted to mix, and the absorbance measured again after 5 min. Then, 25 µl (9.8 U ml^−1^) urease solution was added and the cuvette inverted to mix; the absorbance was measured after 6 min. Finally, 10 µl (6 U ml^−1^) arginase solution was added to the cuvette, the cuvette inverted to mix, and the absorbance measured after 7 min or until absorbance became stable. An internal background control was included in all runs and the reading deducted from all measurements.

#### Measurement of octopine

The content of octopine in the muscle preparations was determined following Chih and Ellington (1983). Octopine content of samples was determined through the action of octopine dehydrogenase (recombinant ODH from *Pecten maximus*, MyBioSource Inc., San Diego, CA, USA) at pH 9.25:

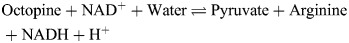
(catalysed by octopine dehydrogenase).

The reaction mixture contained 100 mmol l^−1^ 2-amino-2-methyl-1-propanol (Sigma-Aldrich), 50 mmol l^−1^ hydrazine (Sigma-Aldrich), 12 mmol l^−1^ EDTA (Sigma-Aldrich), 4 mmol l^−1^ NAD^+^ (Cayman Chemical Company, Ann Arbor, MI, USA), and was adjusted to pH 9.2–9.3 using 1 mol l^−1^ hydrochloric acid. Each reaction contained 50 µl of homogenate and was made up to a total volume of 300 µl within a 96 well plate. An internal blank was included containing 50 µl deionised water rather than homogenate; all reactions were initiated by the addition of 0.08 units of ODH. Absorbance was measured prior to enzyme addition, and hourly until stable using a Varioskan Flash microplate reader (ThermoFisher Scientific, Waltham, MA, USA) at 340 nm. NADH absorbs light at 340 nm, with the increase in absorbance being used to determine the content of octopine from the stoichiometry of the reactions.

Measurements of absorbance (*A*) were converted to molar concentrations of metabolites using Beer–Lambert Law:
(2)

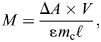
where *V* is the volume (ml), ε is the extinction coefficient at 340 nm (NADH, 6.22 l mmol^−1^ cm^−1^; NADPH, 6.3 l mmol^−1^ cm^−1^), ℓ is the light path (cm) and *m*_c_ is the sample mass (g).

#### Estimation of ATP consumption during exercise

The difference in metabolite concentrations between the worked muscle and the control was used to estimate the total amount of ATP used during the cyclic muscle work contractions. From the stoichiometry of the metabolic pathways, 3 moles of ATP are produced per 2 moles of octopine formed and 1 mole of ATP is produced for every 1 mole of ArgP dephosphorylated. The total ATP equivalents consumed during the cyclical contractions (ATP_total_) was calculated as (Gade, 1981):
(3)

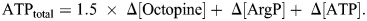


### Contractile efficiency

Contractile efficiency (η_c_) was calculated as the ratio of net mechanical work to metabolic energy expenditure:
(4)

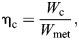
where *W*_c_ is the average net (mechanical) work and *W*_met_ is the cycle average energy input from metabolism. It was assumed that the Gibbs free energy for ATP hydrolysis (Δ*G*_ATP_) was −45 kJ mol^−1^ (Portner et al., 1996), and that recovery during the contractions and in the period between cessation of the cycles and freezing of the tissue was negligible (Melzner et al., 2006).

### Statistical analysis

Data were analysed using IBM SPSS Statistics 24 (International Business Machines Corporation, Armonk, NY, USA). All data were tested for normality and homogeneity prior to statistical testing; data that were not normally distributed were transformed via log transformation to meet the conditions of normality and tested using ANOVA with Tukey *post hoc* tests; data that were non-normally distributed and could not be transformed to meet these assumptions were analysed using non-parametric tests. Comparisons of muscular work and power output were carried out using Kruskal–Wallis tests with *post hoc* Mann–Whitney *U*-tests. Comparisons of the change in metabolite concentrations between cycle frequencies were achieved using ANOVA tests. Comparisons of ATP_total_ and derived parameters between cycle frequencies were determined using ANOVA tests. Statistical significance was defined by a threshold of 0.05; a correction for multiple testing (e.g. Bonferroni) was not applied because of paired tests not influencing other tests (following the recommendations of Armstrong, 2014), and to avoid the risk of increasing type II errors in subsequent tests.

## RESULTS

### Mechanical work and power of the mantle musculature

**Fig. 2.**
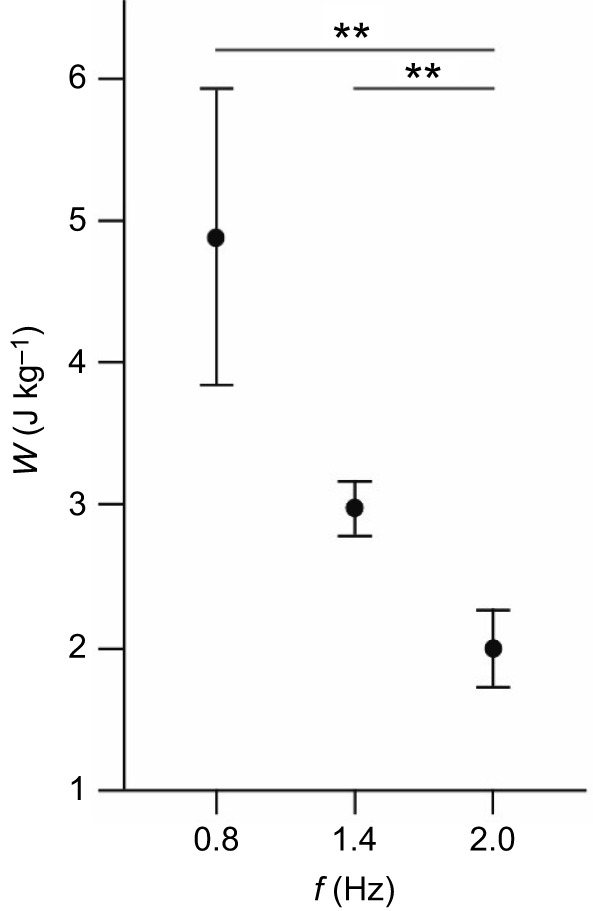
**The cycle average net work (*W*) output of cuttlefish central mitochondria-poor (CMP) muscle during cyclical contractions at a cycle frequency (*f*) of 0.8, 1.4 and 2 Hz.** Data are means±s.e.m., *N*=25 cuttlefish; Mann-Whitney *U post hoc* test, **P<0.01.

**Fig. 3.**
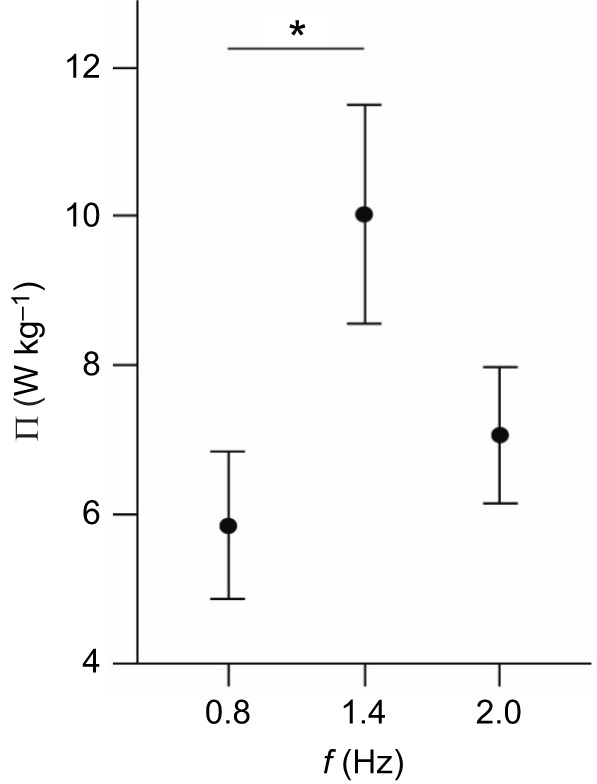
**The average power output (Π) of cuttlefish CMP mantle muscle at 0.8, 1.4 and 2** **Hz.** Data are means±s.e.m., *N*=30 cuttlefish; Tukey *post hoc*, *P<0.05. Power output was averaged over cycles 3 and 4, and combined with data from five additional animals published in Gladman and Askew (2022). See also Fig. S2 where the data from the two studies are presented separately.

The net work per cycle was 4.08±0.26 J kg^−1^ at 0.8 Hz, 2.69±0.11 J kg^−1^ at 1.4 Hz, and 1.99±0.10 J kg^−1^ at 2 Hz. Net work output varied significantly with cycle frequency (*H*=8.43, d.f.=2, *P*=0.015), with significantly higher work at 0.8 Hz than at 2 Hz (*U*=9.9, d.f.=1, *P*=0.006), and significantly higher work at 1.4 Hz than at 2 Hz (*U*=8, d.f.=1, *P*=0.03); no significant differences were found between net work at 1.4 and 0.8 Hz (*U*=1.90, d.f.=1, *P*=0.60; Fig. 2). Power output differed significantly between cycle frequencies (*F=*3.88, d.f.=2, *P*=0.028; Fig. 3), with significantly higher power output at 1.4 Hz than at 0.8 Hz (Tukey *post hoc*, *P*=0.022).

### Changes in metabolite concentrations

**Fig. 4.**
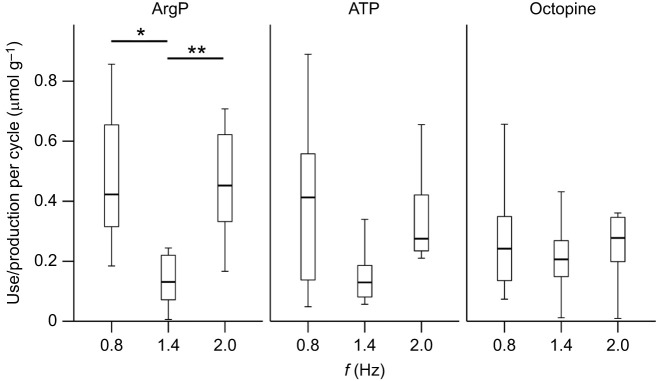
**The concentration of each metabolite used (ArgP and ATP) or produced (octopine) per cycle at each frequency.** Use/production was calculated as the net difference between the control and experimental concentrations, where ATP and ArgP were calculated as control−experimental, and octopine was calculated as experimental−control. The mean (±s.e.m.) concentration of ArgP, ATP and octopine in control preparations was 21.66±7.26, 7.49±1.83 and 3.03±1.08 µmol g^−1^, respectively, at 0.8 Hz (*n*=8); 19.01±0.82, 3.41±0.97 and 3.43±0.43 µmol g^−1^, respectively, at 1.4 Hz (*n*=8); and 21.00±0.41, 6.90±1.04 and 2.79±1.24 µmol g^−1^, respectively, at 2 Hz (*n*=9). Mean (±s.e.m.) concentrations of ArgP, ATP and octopine in experimental preparations were 21.36±0.70, 2.65±0.35 and 4.93±1.42 µmol g^−1^, respectively, at 0.8 Hz (*n*=8); 17.31±1.89, 0.71±0.34 and 8.70±2.41 µmol g^−1^, respectively,  at 1.4 Hz (*n*=8); and 14.71±1.74, 1.79±0.58 and 3.79±0.98 µmol g^−1^, respectively, at 2 Hz (*n*=9). Box plots show median, upper and lower quartiles and 1.5× interquartile range. Tukey *post hoc*, *P<0.05, **P<0.01. *N*=25 cuttlefish.

The octopine formed and ATP utilised per cycle did not vary with cycle frequency (Fig. 4), where 0.34±0.11, 0.20±0.06 and 0.37±0.14 µmol g^−1^ of octopine was formed at 0.8, 1.4 and 2 Hz (*F=*0.37, d.f.=2, *P*=0.69), respectively, and 0.34±0.12, 0.18±0.05 and 0.34±0.06 µmol g^−1^ of ATP was utilised at 0.8, 1.4 and 2 Hz (*F=*3.382, d.f.=2, *P*=0.052), respectively. ArgP utilisation differed significantly between cycle frequencies (Fig. 4), where 0.73±0.35 µmol g^−1^ was utilised at 0.8 Hz, 0.21±0.11 µmol g^−1^ at 1.4 Hz and 0.46±0.07 µmol g^−1^ at 2 Hz (*F=*7.299, d.f.=2, *P*=0.004), with significantly less use at 1.4 Hz than at both 0.8 Hz (Tukey *post hoc*, *P*=0.006) and 2 Hz (Tukey *post hoc*, *P*=0.014). The estimated ATP consumption rate was 1.97±0.40 μmol g^−1^ s^−1^ at 0.8 Hz, 0.49±0.12 μmol g^−1^ s^−1^ at 1.4 Hz and 0.67±0.11 μmol g^−1^ s^−1^ at 2 Hz; assuming a recovery rate of 0.05–0.06 μmol g^−1^ min^−1^ at 15°C, we estimated the percentage recovery to be 0.05±0.01–0.06±0.01% at 0.8 Hz, 0.35±0.20–0.42±0.24% at 1.4 Hz, and 0.14±0.02–0.17±0.02% at 2 Hz.

### Total anaerobic ATP use during cyclical contractions

The total ATP equivalents (ATP_total_; calculated from Eqn 3) consumed per cycle was 1.57±0.32 µmol g^−1^ at 0.8 Hz, 0.69±0.16 µmol g^−1^ at 1.4 Hz and 1.35±0.12 µmol g^−1^ at 2 Hz. ATP use per cycle significantly differed between cycle frequencies (*F=*7.63, d.f.=2, *P*=0.003), with significantly lower ATP consumption at 1.4 Hz than at both 0.8 Hz (Tukey *post hoc*, *P*=0.004) and 2 Hz (Tukey *post hoc*, *P*=0.014). ATP consumption rates were significantly affected by cycle frequency (*F=*16.74, d.f.=2, *P*<0.001), with significantly higher rates at 0.8 Hz than at 1.4 Hz (Tukey *post hoc*, *P*<0.001) and 2 Hz (Tukey *post hoc*, *P*=0.003), where ATP consumption was 1.97±0.40 μmol g^−1^ s^−1^ at 0.8 Hz, 0.49±0.12 μmol g^−1^ s^−1^ at 1.4 Hz and 0.67±0.11 μmol g^−1^ s^−1^ at 2 Hz.

### Contractile efficiency

**Fig. 5.**
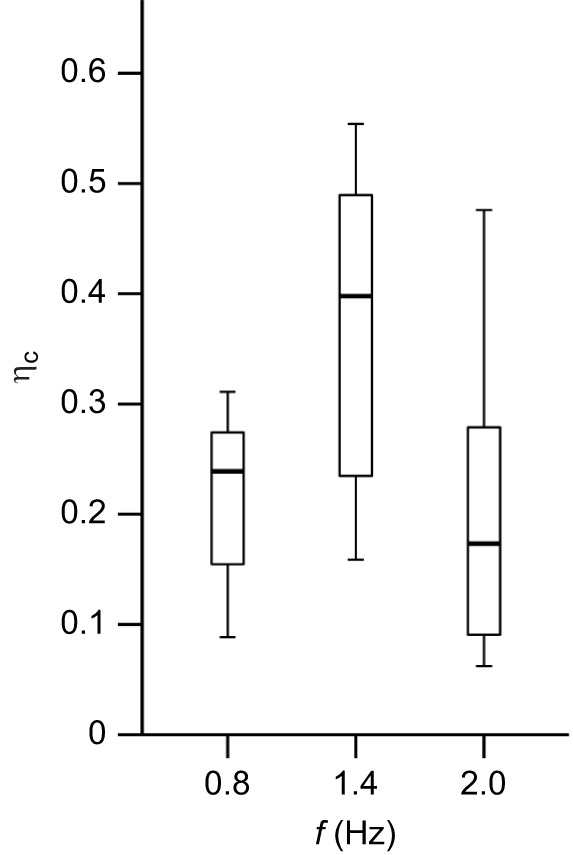
**The contractile efficiency (η­_c_) of cuttlefish mantle muscle during cyclical contractions at 0.8, 1.4 and 2 Hz.** There were no significant differences in η_c_ with cycle frequency (ANOVA *P*>0.05). Box plots show median, upper and lower quartiles and 1.5x interquartile range. *N*=25 cuttlefish, with *n*=8 at 0.8 Hz, *n*=8 at 1.4 Hz and *n*=9 at 2 Hz.

The contractile efficiency (η_c_) of the cuttlefish mantle muscle was 0.27±0.03 at 0.8 Hz, 0.37±0.06 at 1.4 Hz, and 0.20±0.05 at 2 Hz, but there were no significant differences in relation to cycle frequency (*F=*2.61, d.f.=2, *P*=0.098; Fig. 5).

## DISCUSSION

Cuttlefish mantle muscle generated both peak η_c_ and peak power (Gladman and Askew, 2022) at a cycle frequency of 1.4 Hz, which is the frequency used *in vivo* during escape swimming (Gladman and Askew, 2022). At cycle frequencies above and below that used *in vivo*, the muscle generated less power but η_c_ was similar. This is similar to previous work using mouse hindlimb and dogfish myotomal muscle, where the cycle frequency at which peak net power and enthalpy efficiency were obtained was similar, and where efficiency was found to be within 10% of the peak value across a wide range of cycle frequencies [1.5–4 Hz in soleus muscle; 4–14 Hz in extensor digitorum longus muscle (Barclay, 1994); 1.3–3.3 Hz in dogfish white muscle (Curtin and Woledge, 1993b); 0.6–1 Hz in dogfish red muscle (Curtin and Woledge, 1993a)]. Hence, we can conclude that under conditions that yield maximal net power output, cuttlefish CMP muscle fibres operate with maximal contractile efficiency.

The η_c_ value of 0.37 is similar to that measured in scallop adductor muscle (0.30 in *Chlamys hastata* and 0.37 in *Argopecten irradians*) *in vivo* during escape swimming using a similar biochemical estimate of metabolic energy expenditure to that used here (R. L. Marsh, personal communication). The enthalpy efficiency of vertebrate fast-twitch muscles [i.e. the ratio of net mechanical work to the sum of work and heat (enthalpy change); Barclay, 2015] is also similar to that reported in molluscs; for example, in the dogfish (*Scyliorhinus canicula*) white myotomal muscle η_c_ was 0.41 (calculated from net power; Curtin and Woledge, 1993b) and the mouse (*Mus musculus*) extensor digitorum longus muscle η_c_ was 0.34 (calculated from net power; Barclay, 1994). However, the enthalpy efficiency is not equivalent to the contractile efficiency reported here, as part of the free energy change is incorporated into entropy changes (2nd law of thermodynamics; Smith et al., 2005). Assuming ATP is supplied by anaerobic glycolysis with a ratio of total free energy change to enthalpy change of +1.5 (Wilkie, 1960) yields estimates of contractile efficiency of 0.27 and 0.23 for the dogfish and mouse muscles, respectively. Therefore, compared with vertebrate fast-twitch muscles, mollusc muscles appear to have a relatively high contractile efficiency.

η_c_ depends on the value selected for Δ*G*_ATP_. Here, we selected Δ*G*_ATP_ of −45 kJ mol^−1^, which has been measured in squid during submaximal but exhaustive swimming (Portner et al., 1996). In our experiments, muscles were subjected to cyclical contractions in which stimulation timing elicited maximal net work and resulted in a progressive decrease in net work, indicating that the muscles were fatiguing. Therefore, while Δ*G*_ATP_ was higher in squid swimming at slower speeds, we feel that the minimal value of −45 kJ mol^−1^ measured in squid is the most appropriate value to use to estimate η_c_ (Portner et al., 1996).

Over the period during which cyclical work was generated, there was a reduction in the ATP concentration in the muscle. This suggests that ATP was not fully replenished through the anaerobic pathways during this period. The net reduction in available ATP, as well as the build-up of the end-products of fatigue, will limit an animal's ability to sustain locomotion. In cuttlefish, burst responses are not typically used over prolonged periods, instead being a last resort when camouflage is broken by predators (Helmer et al., 2017; Staudinger et al., 2013). These escape responses also involve the release of ink and animal colour changes (Staudinger et al., 2013); these behaviours disrupt pursuit by predators, allowing cuttlefish to minimise the number of jets required to evade predation. The limited capacity of cuttlefish to replenish ATP through anaerobic pathways probably reflects these behaviours, where burst responses are used in a limited manner with ATP recovered following escape. Although the η_c_ of fast-twitch muscle is generally lower than that of slow twitch fibres (Barclay, 1994; Curtin and Woledge, 1993a,b), and the muscles fatigue more rapidly, the ability to replenish ATP rapidly via anaerobic pathways enables these muscles to deliver a burst of high power output, which may be a key determinant of survival.

In the muscle preparations, the radial fibres were not removed. It is possible that these fibres could become activated during the cyclical contractions imposed on the muscle preparation, which could contribute to metabolic energy expenditure. However, in a similar muscle preparation to the one used here, it was demonstrated that sectioning the radial fibres during the dissection disables them and they do not contract (Thompson et al., 2008). In addition, the bulk of the mantle is composed of circular muscle fibres. Therefore, for these two reasons, it is unlikely that the radial fibres contributed significantly to the measured metabolic energy expenditure or impacted upon our calculation of η_c_.

### Overall efficiency of locomotion

At each stage within the energy cascade that results in the transfer of chemical energy into useful hydrodynamic work, energy is lost through the inefficient transduction of energy from one step to the next as well as energy costs associated with processes that are required to sustain muscle contraction but that do not directly contribute to the mechanical output of the muscle (e.g. the costs associated with Ca^2+^ cycling; Fig. 1). The overall efficiency of jet propulsion swimming (η_loco_) is determined by the efficiency of each of these underlying steps; i.e. by η_R_, η_c_ and η_wc_ (Eqn 1; Fig. 1).

The efficiency of mitochondrial oxidative recovery (η_R_) assuming a P/O ratio of 2.5 (Hinkle et al., 1991; reviewed in Hinkle, 2005) is approximately 0.61. In our previous paper, the whole-cycle propulsive efficiency (η_wc_) in juvenile cuttlefish during jet propulsion swimming was found to be ∼0.75 (0.74–0.76 depending on swimming orientation; Gladman and Askew, 2023). Here, the η_c_ of CMP fibres was found to be 0.37, giving an estimate of the overall efficiency of jet propulsion swimming in cuttlefish of 0.17. This estimate of overall efficiency accounts for the mechanical work done during refilling of the mantle cavity, as this is incorporated into the calculation of η_wc_ (see eqn 2 in Gladman and Askew, 2023). The work done to accelerate the water during refilling is only ∼3% of the total hydrodynamic work done in juvenile cuttlefish (Gladman and Askew, 2023) and is thought to be done largely by the release of elastic strain energy from the collagen fibres in the mantle wall that are stretched by the circular muscles during jetting (Curtin et al., 2000). However, it is possible that the radial muscle fibres contribute to the refilling work, which therefore could be done by both the circular and radial muscle fibres. Rather than considering the η_c_ of the radial and circular muscle fibres separately, a single value (0.37 measured for the CMP fibres in this study) was used: we do not know if this is correct for the radial muscle fibres. However, if the mechanical work generated by the circular and radial muscle fibres is considered separately and assuming that the radial fibres do all of the refilling work (which is unlikely; see Curtin et al., 2000), an overall efficiency of 0.17 is still obtained for an assumed radial muscle η_c_ of 0.2 and higher. It seems highly likely that the radial muscle fibre η_c_ lies within this range and therefore that the estimate of overall efficiency of 0.17 is realistic.

Other estimates of the overall efficiency of locomotion range from 0.06 to 0.17 in animal flight [0.06–0.07 in hawkmoths (Casey, 1981; Ellington, 1999; Willmott and Ellington, 1997); 0.17 in starlings (Ward et al., 2001); 0.07–0.10 in cockatiels (Morris and Askew, 2010; Morris et al., 2010); 0.06–0.10 in bats (von Busse et al., 2014)] and from 0.24 to 0.27 in human-powered terrestrial and aquatic pedal vehicles (Capelli et al., 2008). Therefore, contrary to the perception that jet propulsion swimming is inefficient (O'Dor and Webber, 1991), these data suggest that in cuttlefish this mode of locomotion is broadly comparable to the overall efficiency of several other modes of locomotion. We suggest that the high energetic cost of jet propulsion swimming can be attributed to the relatively high mechanical power requirements, rather than by a relatively low efficiency.

## Supplementary Material

10.1242/jexbio.249297_sup1 Supplementary information

## References

[JEB249297C1] Alexander, R. M. (2002). *Principles of Animal Locomotion*. Princeton, NJ, USA: Princeton University Press.

[JEB249297C2] Anderson, E. J. and Grosenbaugh, M. A. (2005). Jet flow in steadily swimming adult squid. *J. Exp. Biol.* 208, 1125-1146. 10.1242/jeb.01507 15767313

[JEB249297C3] Andrews, P. L. R., Darmaillacq, A. S., Dennison, N., Gleadall, I. G., Hawkins, P., Messenger, J. B., Osorio, D., Smith, V. J. and Smith, J. A. (2013). The identification and management of pain, suffering and distress in cephalopods, including anaesthesia, analgesia and humane killing. *J. Exp. Mar. Biol. Ecol.* 447, 46-64. 10.1016/j.jembe.2013.02.010

[JEB249297C58] Armstrong, R. A. (2014). When to use the Bonferroni correction. *Ophthal. Physiol. Optics* 34, 502-508. 10.1111/opo.12131 24697967

[JEB249297C57] Askew, G. N. and Marsh, R. L. (1997). The effects of length trajectory on the mechanical power output of mouse skeletal muscles. *J. Exp. Biol.* 200, 3119-3131. 10.1242/jeb.200.24.3119 9364020

[JEB249297C4] Askew, G. N., Tregear, R. T. and Ellington, C. P. (2010). The scaling of myofibrillar actomyosin ATPase activity in apid bee flight muscle in relation to hovering flight energetics. *J. Exp. Biol.* 213, 1195-1206. 10.1242/jeb.034330 20228356

[JEB249297C5] Barclay, C. J. (1994). Efficiency of fast-twitch and slow-twitch muscles of the mouse performing cyclic contractions. *J. Exp. Biol.* 193, 65-78. 10.1242/jeb.193.1.65 7964400

[JEB249297C6] Barclay, C. J. (2015). Energetics of contraction. *Compr. Physiol.* 5, 961-995. 10.1002/cphy.c140038 25880520

[JEB249297C7] Bartol, I. K., Krueger, P. S., Thompson, J. T. and Stewart, W. J. (2008). Swimming dynamics and propulsive efficiency of squids throughout ontogeny. *Integr. Comp. Biol.* 48, 720-733. 10.1093/icb/icn043 21669828

[JEB249297C8] Bartol, I. K., Krueger, P. S., Stewart, W. J. and Thompson, J. T. (2009a). Hydrodynamics of pulsed jetting in juvenile and adult brief squid *Lolliguncula brevis*: evidence of multiple jet ′modes’ and their implications for propulsive efficiency. *J. Exp. Biol.* 212, 1889-1903. 10.1242/jeb.027771 19483007

[JEB249297C9] Bartol, I. K., Krueger, P. S., Stewart, W. J. and Thompson, J. T. (2009b). Pulsed jet dynamics of squid hatchlings at intermediate Reynolds numbers. *J. Exp. Biol.* 212, 1506-1518. 10.1242/jeb.026948 19411544

[JEB249297C10] Capelli, C., Ardigo, L. P., Schena, F. and Zamparo, P. (2008). Energy cost and mechanical efficiency of riding a human-powered recumbent bicycle. *Ergonomics* 51, 1565-1575. 10.1080/00140130802238614 18803095

[JEB249297C11] Casey, T. M. (1981). A comparison of mechanical and energetic estimates of flight cost for hovering sphinx moths. *J. Exp. Biol.* 91, 117-129. 10.1242/jeb.91.1.117

[JEB249297C12] Cefas (2012). *Sea Temperature and Salinity Trends*, vol. 2016. Centre for Environment, Fisheries and Aquaculture science. https://www.cefas.co.uk/cefas-data-hub/sea-temperature-and-salinity-trends/

[JEB249297C13] Chih, C. P. and Ellington, W. R. (1983). Energy-metabolism during contractile activity and environmental hypoxia in the phasic adductor muscle of the bay scallop *Argopecten irradians concentricus*. *Physiol. Zool.* 56, 623-631. 10.1086/physzool.56.4.30155885

[JEB249297C14] Curtin, N. A. and Woledge, R. C. (1993a). Efficiency of energy-conversion during sinusoidal movement of red muscle-fibers from the dogfish *Scyliorhinus canicula*. *J. Exp. Biol.* 185, 195-206. 10.1242/jeb.185.1.195 1919411

[JEB249297C15] Curtin, N. A. and Woledge, R. C. (1993b). Efficiency of energy-conversion during sinusoidal movement of white muscle-fibers from the dogfish *Scyliorhinus canicula*. *J. Exp. Biol.* 183, 137-147. 10.1242/jeb.183.1.137 1919411

[JEB249297C16] Curtin, N. A., Woledge, R. C. and Bone, Q. (2000). Energy storage by passive elastic structures in the mantle cavity of *Sepia officinalis*. *J. Exp. Biol.* 203, 869-878. 10.1242/jeb.203.5.869 10667969

[JEB249297C17] Ellington, C. P. (1999). The novel aerodynamics of insect flight: applications to micro-air vehicles. *J. Exp. Biol.* 202, 3439-3448. 10.1242/jeb.202.23.3439 10562527

[JEB249297C18] Feala, J. D., Coquin, L., Mcculloch, A. D. and Paternostro, G. (2007). Flexibility in energy metabolism supports hypoxia tolerance in *Drosophila* flight muscle: metabolomic and computational systems analysis. *Mol. Syst. Biol.* 3, 99. 10.1038/msb4100139 17437024 PMC1865581

[JEB249297C19] Gade, G. (1981). Energy-production during swimming in the adductor muscle of the bivalve *Lima hians* - comparison with the data from other bivalve mollusks. *Physiol. Zool.* 54, 400-406. 10.1086/physzool.54.4.30155832

[JEB249297C20] Gäde, G., Weeda, E. and Gabbott, P. A. (1978). Changes in the level of octopine during the escape responses of the scallop, *Pecten maximus* (L.). *J. Comp. Physiol.* 124, 121-127. 10.1007/BF00689172

[JEB249297C21] Gladman, N. W. (2018). The energetics and mechanics of jet propulsion swimming in European common cuttlefish (Sepia officinalis). *PhD thesis*, University of Leeds. 10.1242/jeb.246225 PMC10560557 37655637

[JEB249297C22] Gladman, N. W. and Askew, G. N. (2022). The mechanical properties of the mantle muscle of European cuttlefish (*Sepia officinalis*). *J. Exp. Biol.* 225, jeb244977. 10.1242/jeb.244977 36416079 PMC10112868

[JEB249297C23] Gladman, N. W. and Askew, G. N. (2023). The hydrodynamics of jet propulsion swimming in hatchling and juvenile European common cuttlefish, *Sepia officinalis*. *J. Exp. Biol.* 226, jeb246225. 10.1242/jeb.246225 37655637 PMC10560557

[JEB249297C24] Grieshaber, M. and Gäde, G. (1976). The biological role of octopine in the squid, *Loligo vulgaris* (Lamarck). *J. Comp. Physiol.* 108, 225-232. 10.1007/BF00691671

[JEB249297C25] Helmer, D., Geurten, B. R. H., Dehnhardt, G. and Hanke, F. D. (2017). Saccadic movement strategy in common cuttlefish (*Sepia officinalis*). *Front. Physiol.* 7, 660. 10.3389/fphys.2016.00660 28105017 PMC5214429

[JEB249297C26] Hinkle, P. C. (2005). P/O ratios of mitochondrial oxidative phosphorylation. *Biochim.Biophys. Acta Bioenerg.* 1706, 1-11. 10.1016/j.bbabio.2004.09.004 15620362

[JEB249297C27] Hinkle, P. C., Kumar, M. A., Resetar, A. and Harris, D. L. (1991). Mechanistic stoichiometry of mitochondrial oxidative-phosphorylation. *Biochemistry* 30, 3576-3582. 10.1021/bi00228a031 2012815

[JEB249297C28] Holt, N. C. and Azizi, E. (2014). What drives activation-dependent shifts in the force-length curve. *Biol. Lett.* 10, 20140651. 10.1098/rsbl.2014.0651 25252838 PMC4190969

[JEB249297C29] Josephson, R. K. (1985). Mechanical power output from striated-muscle during cyclic contraction. *J. Exp. Biol.* 114, 493-512. 10.1242/jeb.114.1.493

[JEB249297C30] Kier, W. M. and Curtin, N. A. (2002). Fast muscle in squid (*Loligo pealei*): contractile properties of a specialized muscle fibre type. *J. Exp. Biol.* 205, 1907-1916. 10.1242/jeb.205.13.1907 12077167

[JEB249297C31] Lowry, O. H. and Passonneau, J. V. (1972). *A Flexible System of Enzymatic Analysis*. Academic Press.

[JEB249297C32] Maertens, A. P., Triantafyllou, M. S. and Yue, D. K. P. (2015). Efficiency of fish propulsion. *Bioinspir. Biomim.* 10, 046013. 10.1088/1748-3190/10/4/046013 26226349

[JEB249297C33] Melzner, F., Bock, C. and Portner, H. O. (2006). Critical temperatures in the cephalopod Sepia officinalis investigated using in vivo P-31 NMR spectroscopy. *J. Exp. Biol.* 209, 891-906. 10.1242/jeb.02054 16481578

[JEB249297C34] Milligan, B. J., Curtin, N. A. and Bone, Q. (1997). Contractile properties of obliquely striated muscle from the mantle of squid (*Alloteuthis subulata*) and cuttlefish (*Sepia officinalis*). *J. Exp. Biol.* 200, 2425-2436. 10.1242/jeb.200.18.2425 9320349

[JEB249297C35] Mira De Orduña, R. (2001). Quantitative determination of l-arginine by enzymatic end-point analysis. *J. Agric. Food Chem.* 49, 549-552. 10.1021/jf000522y 11261990

[JEB249297C36] Morris, S. and Adamczewska, A. M. (2002). Utilisation of glycogen, ATP and arginine phosphate in exercise and recovery in terrestrial red crabs, *Gecarcoidea natalis*. *Comp. Biochem. Physiol. A: Mol. Integr. Physiol.* 133, 813-825. 10.1016/S1095-6433(02)00217-9 12443937

[JEB249297C37] Morris, C. R. and Askew, G. N. (2010). The mechanical power output of the pectoralis muscle of cockatiel (*Nymphicus hollandicus*): the in vivo muscle length trajectory and activity patterns and their implications for power modulation. *J. Exp. Biol.* 213, 2770-2780. 10.1242/jeb.035691 20675547

[JEB249297C38] Morris, C. R., Nelson, F. E. and Askew, G. N. (2010). The metabolic power requirements of flight and estimations of flight muscle efficiency in the cockatiel (*Nymphicus hollandicus*). *J. Exp. Biol.* 213, 2788-2796. 10.1242/jeb.035717 20675549

[JEB249297C39] Neil, T. R. and Askew, G. N. (2018). Swimming mechanics and propulsive efficiency in the chambered nautilus. *R. Soc. Open Sci.* 5, 170467. 10.1098/rsos.170467 29515819 PMC5830708

[JEB249297C40] O'Dor, R. K. and Webber, D. M. (1991). Invertebrate athletes - trade-offs between transport efficiency and power-density in cephalopod evolution. *J. Exp. Biol.* 160, 93-112. 10.1242/jeb.160.1.93

[JEB249297C41] Portner, H. O., Finke, E. and Lee, P. G. (1996). Metabolic and energy correlates of intracellular pH in progressive fatigue of squid (*L. brevis*) mantle muscle. *Am. J. Physiol. Regul. Integr. Comp. Physiol.* 271, R1403-R1414. 10.1152/ajpregu.1996.271.5.R1403 8945980

[JEB249297C42] Preuss, T., Lebaric, Z. N. and Gilly, W. F. (1997). Post-hatching development of circular mantle muscles in the squid *Loligo opalescens*. *Biol. Bull.* 192, 375-387. 10.2307/1542747 9212445

[JEB249297C43] Rosenbluth, J., Szent-Gyorgyi, A. G. and Thompson, J. T. (2010). The ultrastructure and contractile properties of a fast-acting, obliquely striated, myosin-regulated muscle: the funnel retractor of squids. *J. Exp. Biol.* 213, 2430-2443. 10.1242/jeb.037820 20581273 PMC2892422

[JEB249297C44] Roumbedakis, K., Alexandre, M. N., Puch, J. A., Martins, M. L., Pascual, C. and Rosas, C. (2020). Short and long-term effects of anesthesia in *Octopus maya* (Cephalopoda, Octopodidae) Juveniles. *Front. Physiol.* 11, 697. 10.3389/fphys.2020.00697 32695019 PMC7338579

[JEB249297C45] Smith, N. P., Barclay, C. J. and Loiselle, D. S. (2005). The efficiency of muscle contraction. *Prog. Biophys. Mol. Biol.* 88, 1-58. 10.1016/j.pbiomolbio.2003.11.014 15561300

[JEB249297C46] Staudinger, M. D., Buresch, K. C., Mathger, L. M., Fry, C., Mcanulty, S., Ulmer, K. M. and Hanlon, R. T. (2013). Defensive responses of cuttlefish to different teleost predators. *Biol. Bull.* 225, 161-174. 10.1086/BBLv225n3p161 24445442

[JEB249297C47] Storey, K. B. and Storey, J. M. (1979). Octopine metabolism in the cuttlefish, *Sepia officinalis* - octopine production by muscle and its role as an aerobic substrate for non-muscular tissues. *J. Comp. Physiol.* 131, 311-319. 10.1007/BF00688806

[JEB249297C48] Thompson, J. T., Szczepanski, J. A. and Brody, J. (2008). Mechanical specialization of the obliquely striated circular mantle muscle fibres of the long-finned squid *Doryteuthis pealeii*. *J. Exp. Biol.* 211, 1463-1474. 10.1242/jeb.017160 18424680

[JEB249297C49] Thompson, J. T., Shelton, R. M. and Kier, W. M. (2014). The length-force behavior and operating length range of squid muscle vary as a function of position in the mantle wall. *J. Exp. Biol.* 217, 2181-2192. 10.1242/jeb.083907 24675565

[JEB249297C50] Thompson, J. T., Taylor-Burt, K. R. and Kier, W. M. (2023). One size does not fit all: diversity of length–force properties of obliquely striated muscles. *J. Exp. Biol.* 226, jeb244949. 10.1242/jeb.244949 36633589 PMC10658899

[JEB249297C51] Von Busse, R., Waldman, R. M., Swartz, S. M., Voigt, C. C. and Breuer, K. S. (2014). The aerodynamic cost of flight in the short-tailed fruit bat (*Carollia perspicillata*): comparing theory with measurement. *J. R. Soc. Interface* 11, 20140147. 10.1098/rsif.2014.0147 24718450 PMC4006254

[JEB249297C52] Ward, S., Moller, U., Rayner, J. M. V., Jackson, D. M., Bilo, D., Nachtigall, W. and Speakman, J. R. (2001). Metabolic power, mechanical power and efficiency during wind tunnel flight by the European starling *Sturnus vulgaris*. *J. Exp. Biol.* 204, 3311-3322. 10.1242/jeb.204.19.3311 11606605

[JEB249297C53] Wells, M. J. and O'dor, R. K. (1991). Jet propulsion and the evolution of the cephalopods. *Bull. Mar. Sci.* 49, 419-432.

[JEB249297C54] Wilkie, D. R. (1960). Thermodynamics and the interpretation of biological heat measurements. *Prog. Biophys. Mol. Biol.* 10, 259-298. 10.1016/S0096-4174(18)30192-6 13785302

[JEB249297C55] Willmott, A. P. and Ellington, C. P. (1997). The mechanics of flight in the hawkmoth *Manduca sexta*. 2. Aerodynamic consequences of kinematic and morphological variation. *J. Exp. Biol.* 200, 2723-2745. 10.1242/jeb.200.21.2723 9418030

[JEB249297C56] Zullo, L., Di Clemente, A. and Maiole, F. (2022). How octopus arm muscle contractile properties and anatomical organization contribute to arm functional specialization. *J. Exp. Biol.* 225, jeb243163. 10.1242/jeb.243163 35244172

