# Supplementary material for: The contractile efficiency of the mantle muscle of European common cuttlefish (Sepia officinalis) during cyclical contractions
Source: J Exp Biol. 2024 Nov 8;227(21):jeb249297. doi: 10.1242/jeb.249297 (PMC11583979; doi:10.1242/jeb.249297)
Supplement: Supplementary information [file jexbio-227-249297-s1.pdf]

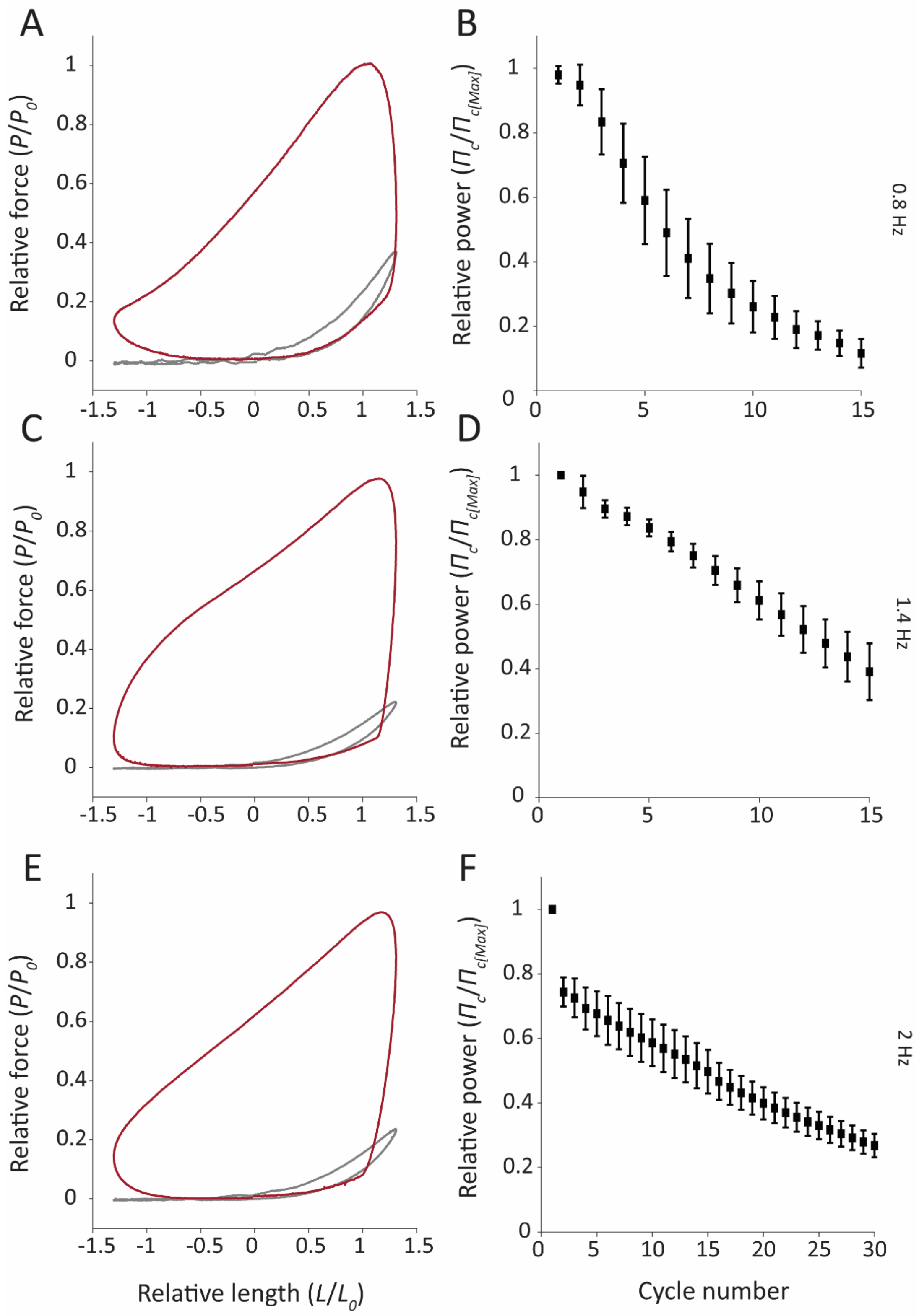

Fig. S1. Example cyclic muscle properties of cuttlefish CMP muscle. (A, C and F) show example stimulated (red) and unstimulated (grey) workloops at (A) 0.8 Hz, (C) 1.4 Hz, and (E) 2 Hz. Records are normalised to the peak force and L_0. (B, D and F) show the mean (± S.D.) per cycle power output of cuttlefish CMP muscle bundles at (B) 0.8 Hz, (D) 1.4 Hz and (F) 2 Hz.

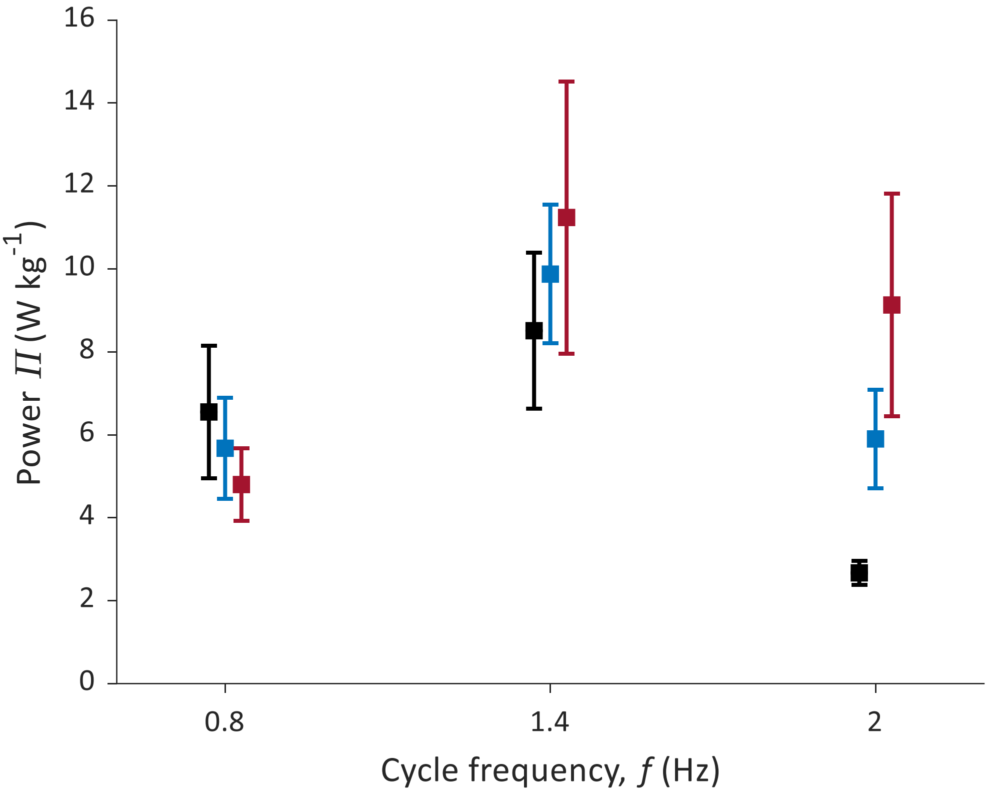

Fig. S2. Comparison of the mean (± sem) power output at 0.8, 1.4 and 2 Hz from cycles 3 and 4. The means from this study (black, N = 25), Gladman & Askew (2022; red, N = 5), and the combined dataset (blue, N = 30) are shown. Power output did not differ significantly 0.8 Hz ($t = 0.60$, $df = 10$, $p = 0.60$) or 1.4 Hz ($t = 0.78$, $df = 11$, $p = 0.45$). Power at 2 Hz was however significantly lower in this study than in Gladman & Askew (2022; $t = 3.49$, $df = 10$, $p = 0.006$).

Table S1. Stimulus train duration and phase of stimulation (relative to peak length) used to elicit peak power output of cuttlefish muscle (following Gladman & Askew, 2022).

Cycle frequency (Hz)	Train duration (ms)	Phase (ms)
0.8	475	-100
1.4	200	-100
2.0	65	-100

Journal of Experimental Biology • Supplementary information
